# Ogilvie syndrome after cesarean section

**DOI:** 10.1007/s00404-023-07052-2

**Published:** 2023-05-08

**Authors:** V. Rothfuss, C. Reisenauer, C. Bachmann

**Affiliations:** Department of Women’s Health Tübingen, Eberhard Karls University Tübingen, 72076 Tübingen, Germany

## Presentation

**Fig. 1:**
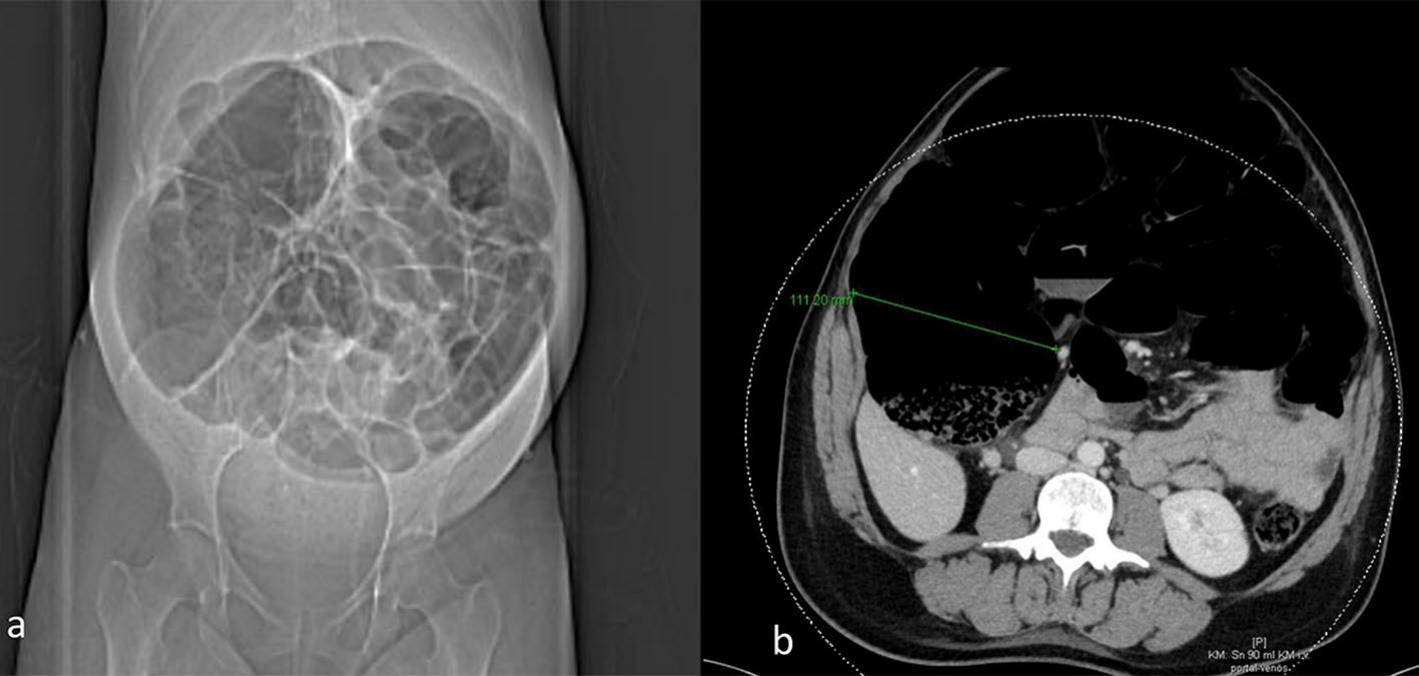
**a** Abdominal X-ray. Extremely distended abdomen with signs of massive dilatation of the colon. **b** CT massive dilatation of colon especially caecum up to 11 cm (marked with a green arrow)

An elective cesarean section (CS) was performed in a 29-year-old primiparous woman, with no prior surgeries, at 37 weeks of gestation in monochorionic diamniotic twins. On the first postoperative day, the patient complained of severe generalized abdominal pain with no improvement after intravenous analgesics and a distended abdomen (Fig. 1a). The biochemistry demonstrated a C-reactive protein (CRP) of 5.33 mg/dl, and the examination revealed a distended abdomen (Fig. 1a) with normal bowel movements. The abdominal ultrasound was diagnostically inconclusive due to bowel dilatation with no evidence of incarceration or hematoma. Computed tomography scan (CT) showed a severe ileus with a caecum diameter up to 12 cm as well as dilated small intestine loops and a dilated transverse, descending and sigmoid colon (Fig. 1b). No other pathologies were found. A laparotomy was carried out and the decompression of the dilated bowel was successfully performed by inserting a catheter through the amputated appendix.

There were no other pathologies or a mechanical cause of ileus. The patient recovered well.

## Discussion

Ogilvie syndrome or acute colonic pseudo-obstruction is a rare but severe complication after abdominal surgeries including cesarean section [1], trauma or severe burning. It is characterized by a non-obstructed colon consistent with a paralytic ileus. The caecal and colon dilatation progresses rapidly and if left untreated bowel ischaemia and perforation can occur causing a high mortality rate of up to 40% [2]. Diagnosis is based on clinical or/and CT findings. [3]. Management depends on patient’s clinical presentation and the caecal diameter on CT imaging and comprises a conservative, pharmacologic treatment followed by a decompression of the colon through a colonoscopy or a surgical intervention [4–6]. The risk of bowel perforation increases with persistence of a caecal diameter > 12 cm [1]. The CS-related incidence of Ogilvie’s syndrome is 1/800 [7].

## Data Availability

Data are available from the author.
